# A Comprehensive Review of the Role of GLP-1 Agonists in Weight Management and Their Effect on Metabolic Parameters Such as Blood Glucose, Cholesterol, and Blood Pressure

**DOI:** 10.7759/cureus.76519

**Published:** 2024-12-28

**Authors:** Ushna Gul, Thandar Aung, Mehwish Martin, Daanyal N Farrukh, Pari C Shah, Zeenia S Lovely, Esaúl Marroquín León, Mohamed Alansaari, Shriya Maini, Muddasir Mohammed Fariduddin, Ashraf Ullah, Zahra Nazir

**Affiliations:** 1 Internal Medicine, Khyber Medical College, Peshawar, PAK; 2 Accident and Emergency, St. Ann's Bay Hospital, St. Ann's Bay, JAM; 3 Internal Medicine, Icahn School of Medicine at Mount Sinai, New York, USA; 4 Kinesiology, McMaster University, Hamilton, CAN; 5 Family Medicine, Northeast Ohio Medical University, Xenia, USA; 6 Emergency, Kerala University of Health and Sciences, Cochin, IND; 7 Medicine, Facultad de Medicina, Universidad Westhill, Ciudad de México, MEX; 8 Internal Medicine, Royal College of Surgeons in Ireland (RCSI) University of Medicine and Health Sciences, Dublin, IRL; 9 Internal Medicine, Dayanand Medical College and Hospital, Punjab, IND; 10 Internal Medicine, Ayaan Institute of Medical Sciences, Hyderabad, IND; 11 Internal Medicine, Mayo Hospital, Lahore, PAK; 12 Internal Medicine, Combined Military Hospital, Quetta, PAK

**Keywords:** blood pressure, cholesterol, diabete mellitus, glp-1 receptor agonists, obesity

## Abstract

Glucagon-like peptide-1 receptor agonists (GLP-1 RAs) have been developed to manage type 2 diabetes mellitus. Although, in the last 10 years, the use of GLP-1 RAs, especially semaglutide and liraglutide, has increased, its clinical implications and how it affects metabolic parameters have yet to be fully consolidated.

This narrative review explores the metabolic effects of GLP-1 RAs in weight management, blood glucose, cardiovascular health, lipid profiles, and blood pressure. Data were collected by comparing GLP-1 RAs, such as semaglutide, liraglutide, tripeptide, and exenatide, as well as comparing them to a baseline treatment group.

GLP-1 RAs have shown consistent results in managing blood glucose levels by lowering HbA1c with minimal hypoglycemic risk and increasing insulin production and synthesis. GLP-1 RAs have been found to improve overall cardiovascular health and reduce major adverse cardiovascular events (MACE) by improving the endothelial function of the vasculature and lowering ANP (atrial natriuretic peptide) production, leading to reduced blood pressure. In addition to the cardiovascular benefits, GLP-1 RAs have a varying effect on lipid profiles, finding statistically significant results for low-density lipoprotein cholesterol levels. In conjunction with all the effects, GLP-1 RAs have been found to lower weight and aid in weight management.

## Introduction and background

Glucagon-like peptide-1 receptor agonists (GLP-1 RAs), especially semaglutide (famously known as Ozempic® or Wegovy®), have become very popular in recent years for weight loss, even though their initial indication is for the management of the patient with diabetes mellitus. GLP-1 RAs were created after endogenous glucagon-like peptide-1 (GLP-1) was discovered in the 1980s by Jens Juul Host and Joel Habener [1,2]. As part of the incretin hormones, GLP-1 has three known mechanisms of action: activating glucose-dependent insulin secretions, thereby reducing plasma glucose, suppressing glucagon secretion, and delaying gastric emptying [3]. However, endogenous GLP-1 can be inactivated by dipeptidyl peptidase-4 (DPP-4) [4]. Similar compounds that could bind to GLP-1 receptors but resist inactivation by DDP-4 were researched and found to help treat type 2 diabetes mellitus (T2DM). These compounds have similar amino acid sequences compared to endogenous GLP-1, with small alterations, a free fatty acid side chain that binds to albumin, explicitly seen in liraglutide and semaglutide, that alter their pharmacokinetic properties (e.g., increased half-life and duration of action) [3].

Synthetic exendin-4, derived from the peptide exendin-4 discovered in a venomous lizard's saliva, was homologous to human GLP-1 [4,5] and was named exenatide. Thus, GLP-1 RAs were first introduced in 2005 when the FDA approved exenatide for glycemic control in T2DM. Since then, GLP-1 RAs have become a cornerstone treatment option for T2DM, as it causes a mean 0.8-1.5% reduction in HbA1c with the minimum associated risk of hypoglycemia. It is primarily used as subcutaneous injections and oral formulations [6,7]. Additionally, these GLP-1 RAs have been explored as an effective means of weight loss, with the STEP clinical trials showing a mean of 5% weight loss at the end of 68 weeks of treatment. Specifically, the STEP-4 trials recorded a 10.6% weight loss with semaglutide [8,9]. Following this, the FDA-approved Wegovy® (semaglutide) was given as a treatment for weight loss in patients with a body mass index (BMI) of 27 kg/m2 or higher [10].

Patients with T2DM are also at an increased risk of myocardial infarction, stroke, and other major adverse cardiac events (MACE) [11]. GLP-1 RAs have been emerging as an effective means of mitigating this risk. They work by lowering arterial blood pressure, which, in turn, reduces the risk of MACE [7,11]. Various clinical trials have reported these findings, which are discussed further in our study [11-14]. Given the variety of GLP-1 RAs, several meta-analyses have also compared the different effects of these medications on lipid profile, with a consensus showing a decrease in low-density lipoprotein (LDL) and triglycerides, with some GLP-1 RAs, such as exenatide, increasing the high-density lipoprotein (HDL) levels [14-17].

All these emerging benefits have made GLP-1 RAs an important pharmacological drug. In this narrative review, we have explored the varying effects of these agents on essential metabolic parameters such as blood glucose, cardiovascular health, weight management, blood pressure, and lipid profile in both diabetic and non-diabetic patients. Although ample clinical trials and meta-analyses have been conducted to highlight these limits separately, more work is needed to narrate all these metabolic parameters under one umbrella. This narrative review aims to explore the role of GLP-1 RAs in weight management and cardiovascular health, and how they affect metabolic parameters such as blood glucose, lipid profiles, and blood pressure.

## Review

The following discussion will delve into further details regarding the mechanism of action of GLP-RAs and their evolution over the past couple of years. Since GLP-RAs were created primarily for the treatment of T2DM, their effect on blood glucose will be discussed first, followed by their effect on weight management and obesity. The GLP-RAs have also been found to affect blood pressure and cholesterol, benefiting cardiovascular health in general. Finally, the limitations and long-term adverse effects will be explained, and the future potential of GLP-RAs will also be addressed.

**Figure 1:**
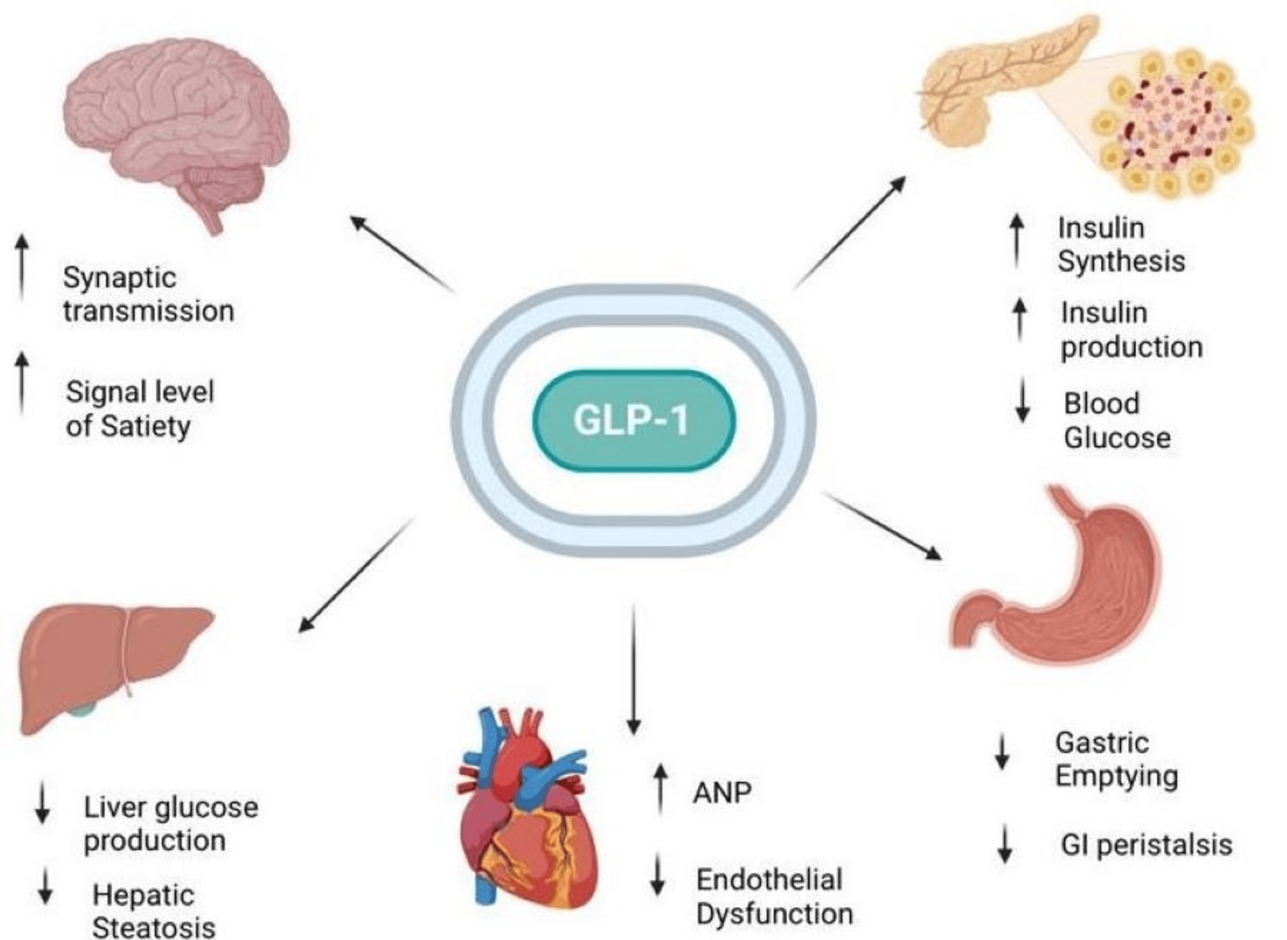
The effects of GLP-1 agonists on targeted tissues Source reference: [2,3] GLP-1, glucagon-like peptide-1

GLP-1 RAs have numerous pharmacological effects on the management of T2DM. The effects of exogenous GLP-1 after administration to T2DM patients show improved insulin sensitivity, decreased glucagon concentration, slowed gastric emptying, increased satiety, decreased fatty acid concentration, lowered body weight, and overall decreased hemoglobin A1c (HBA1c) levels. Figure 1 depicts the action of GLP-1 RAs on target tissues.

Since the first class GLP-1 agent exenatide was approved several years ago, multiple GLP-1 RAs have become readily available in the United States. 

Effects of GLP-1 RAs on blood glucose

With the present data available, it is well established that GLP-1 RAs are effective medications for lowering blood glucose levels and managing T2DM. In a recent study published by Nauck et al., all GLP-1 RAs have a common mechanism of action in terms of controlling blood glucose levels, i.e., increased production of insulin during hyperglycemic states, suppressing glucagon secretion, delayed gastric emptying, preventing postprandial glucose spikes, and lowering caloric intake and BMI [3]. Moreover, their study also provided significant inferences comparing the effectiveness of glycemic control for overnight and fasting glucose levels for short- and long-acting GLP-1 RAs [3].

Short-acting GLP-1 RAs (e.g., exenatide, lixisenatide) were found to have a reduced impact on overnight and fasting blood glucose levels, while long-acting GLP-1 RAs (liraglutide, exenatide once weekly, dulaglutide, and semaglutide) were found to have an increased effect and better coverage on overnight and fasting blood glucose and HbA1c levels [18]. Given their effectiveness in controlling blood glucose and HbA1c levels, with additional weight loss benefits and minimal intrinsic risk of hypoglycemic episodes, GLP-1 RAs are now considered the first-line injectables for optimal glucose control in diabetic patients even before insulin treatment [18].

According to the American Diabetes Association, the first-line glycemic control therapy focuses on comorbidities, lifestyle modification, and medications such as metformin and combination therapy with GLP-1 RAs and sodium-glucose cotransporter-2 (SGLT-2) inhibitors [18]. Clinicians should recommend a patient-centered approach to choosing the appropriate medications for blood glucose management. In recent years, diabetes treatment has focused more on increasing hormones. Incretin is a hormone secreted by the intestinal mucosa when ingested nutrients arrive in the intestine. It enhances the effect of glucose-mediated insulin secretion to lower blood glucose levels. The effect of insulin secretion due to oral glucose consumption is higher than intravenous glucose infusion and is called the “incretin effect.” Two incretin hormones, glucagon-like peptide and glucose-dependent insulinotropic polypeptide, have been studied more in recent years due to their glucose-lowering effects for T2DM. The increase in release is decreased in T2DM, which deteriorates the GLP-1 effect, which impairs insulin secretion and results in insulin resistance and hyperglycemia. The administration of GLP-1 agonists stimulates the GLP-1 receptors, increasing the insulin secretion to both oral and intravenous glucose [19].

The HbA1c target of <53 mmol/mol (<7.0%) or ≤47.5 mmol/mol (≤6.5%) was achieved in a higher proportion of patients treated with incretin-based glucose-lowering medications compared to basal insulin by 18.5% and 23.0%, respectively. This was not significant when comparing with short-acting GLP-1 RAs and basal insulin treatment for both targets but significant when comparing long-acting GLP-1 RAs and basal insulin (Odds ratio 5.42 and 5.43, respectively; both p-values <0.001) [20]. Based on non-overlapping 95% CIs, the effects of tripeptide were significantly greater than those of long-acting GLP-1 RAs [20].

Effect of GLP-1 RAs on weight management

Obesity is a complex, neuro-metabolic disease in which too much fat accumulates in an individual’s body, potentially causing many adverse effects on the body and associated comorbidities [21]. These associated comorbidities include T2DM, hypertension, cardiovascular disease, steatotic liver disease (SLD), ventilatory dysfunction, arthrosis, depression, and increased morbidity and mortality risk. A BMI of 30 kg/m^2^ or higher classifies as obesity, specifically class 1 obesity. Traditionally, obesity has been controlled with diet, exercise, behavior modification, and bariatric surgery. However, in recent years, there has been a significant rise in the use of antiobesity medications such as GLP-1 RAs with promising results in weight loss and management [22,23].

GLP-1 RAs have been widely researched and used as antiobesity drugs in patients with or without diabetes. Using search engines such as PubMed, 3,730 articles were found to be on GLP-1 RAs alone in 10 years, with 1,194 articles on their role in weight management and control of obesity. The popular GLP-1 RAs used for this purpose are once or biweekly injectables, such as semaglutide, liraglutide, and tirzepatide. As per Boje et al., trials with semaglutide have shown consistent superiority in providing adequate glycemic and weight control compared to other oral anti-diabetic agents and basal insulin [24]. In this phase III clinical trial, a total of 8,416 patients were enrolled in the SUSTAIN program (Semaglutide Unabated Sustainability in Treatment of Type 2 diabetes) divided into seven trials, where the effectiveness of semaglutide (weekly dosing schedule) was compared individually against the effectiveness of placebo in monotherapy (two antidiabetic agents) and, in the same way, against insulin glargine, dulaglutide, exenatide, and sitagliptin. At the end of 30 to 52 weeks of the clinical trial, a maximum weight loss of 4.6 kg (semaglutide 0.5 mg) and 6.5 kg (semaglutide 1.0 mg) was achieved. The results were significantly greater than those achieved with the placebo, and since it was compared with other drugs of the same class, it is known that semaglutide also obtained better results than its analogs such as exenatide or dulaglutide [24].

Furthermore, to understand and capture the results of the use of semaglutide, emphasizing weight loss and obesity management, it can be stated that, with correct adherence to the patient's pharmacological treatment and healthier lifestyle changes in patients with a BMI greater than 30 kg/m^2^, a reduction of approximately 13.8% of body weight can be obtained after 52 weeks; however, around 30% of patients in this clinical trial lost more than 20%. Nevertheless, more evidence is still required to standardize the results described in these studies [24].

**Figure 2:**
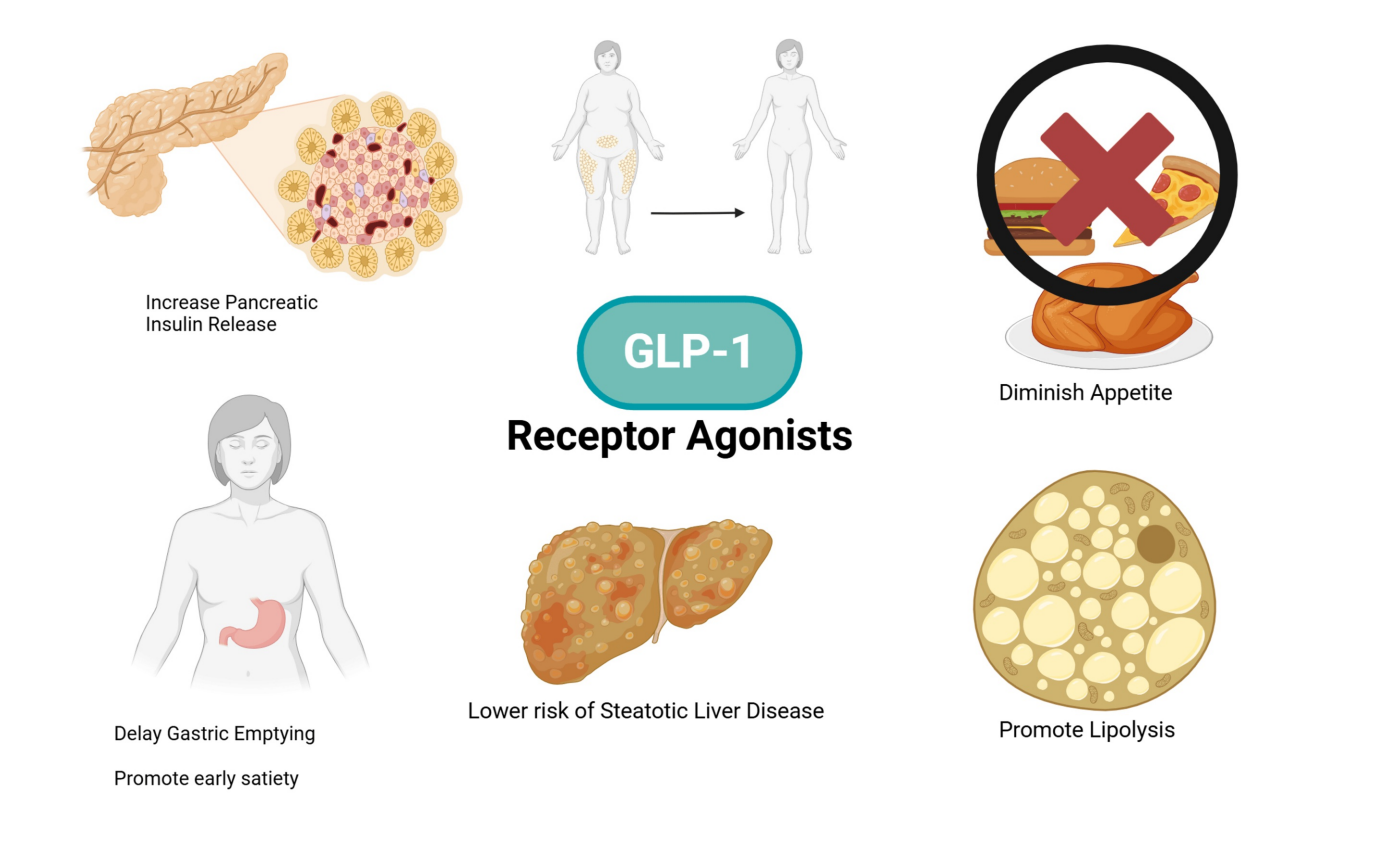
Mechanism of action of GLP-1 receptor agonists Source reference: [24,25] GLP-1, glucagon-like peptide-1

The choices of antiobesity drugs are expanding, with the most promising results achieved with the increasing analogs. More data and long-term studies are needed to determine the side effect profile and the long-term effects of these medications on the human body [25]. Figure 2 demonstrates pathways by which GLP-1 RAs cause weight loss, reduce blood glucose and cholesterol levels, and decrease the risk of SLD.

Efficacy of GLP-1 RAs in improving cardiovascular parameters

Cardiovascular disease (CVD) is one of the most concerning ailments affecting people worldwide, with an estimated 48.6% of Americans suffering from heart-related illnesses [26]. Patients suffering from obesity and diabetes are at a higher risk of these events. With modern research, a growing body of evidence highlights the possible use of GLP-1RAs to reduce major adverse cardiovascular events (MACE) and improve overall outcomes in diabetic or obese patients. GLP-1 RAs have been effective in decreasing the incidence of myocardial infarctions, hospitalizations, and non-fatal strokes, as evidenced by the PIONEER, SUSTAIN, and LEADER trials over the years [27-30].

Over the years, several theories have emerged demonstrating the possible mechanism through which GLP-RAs improve cardiovascular health in the circulatory system. Rizzo et al. summarize that hyperglycemia causes insulin resistance, producing dyslipidemia, free radicals, and endothelial dysfunction. This can lead to a condition of diabetic cardiomyopathy with deposition of palmitate in the heart walls [31], inducing apoptosis. GLP-RAs have been shown to stop this process by increasing b-catenin signaling. They reduce these atherogenic lipoproteins, improve endothelial vasodilation, and reduce inflammation, as evidenced by reduced CRP levels [28,32,33]. In diabetic patients treated with GLP-1 RAs, better blood circulation and improved endothelial function have been recorded [34,35]. Among all the different GLP-RAs, Liraglutide has been shown to slow down the plaque establishment process and decrease carotid intimal thickness, as reviewed by Patti et al. [36]. Similarly, a treatment course of Semaglutide for 4 months showed a significant reduction of 13% in the carotid intima thickness. This supported the theory that GLP-RAs lower atherosclerosis markers, which lessens MACE incidence [32,34,36].

**Figure 3:**
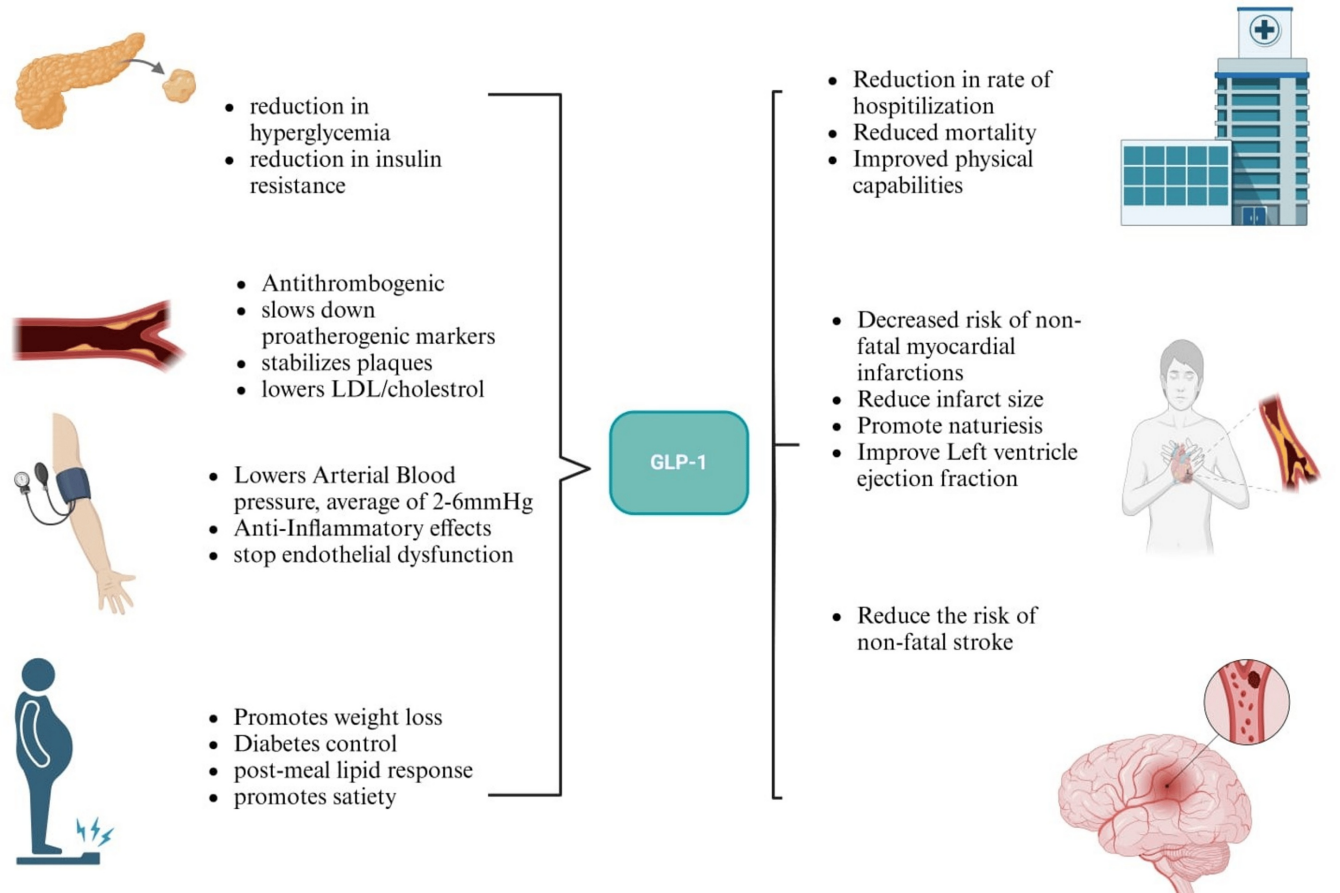
Mechanisms through which GLP-1 RAs have beneficial effects on the cardiovascular system and the documented benefits in cardiovascular outcome trials Source reference: [32,37,38] GLP-1 RAs, glucagon-like peptide-1 receptor agonists

Figure 3 demonstrates the mechanisms through which GLP-RAs have beneficial effects on the cardiovascular system and the documented benefits in cardiovascular outcome trials (CVOTs) [32,37,38].

Efficacy of GLP-1 RAs in reducing MACE

A comprehensive meta-analysis conducted by Sattar et al. studying the effects of GLP-1 RAs on the heart showed a promising 12% reduction in mortality, with a 14% decrease in the risk of cardiovascular events and an 11% decrease in the rate of hospital admissions due to heart failure. Similarly, the analysis of various clinical trials suggests an 8% decreased risk of nonfatal myocardial infarction in patients using semaglutide compared to placebo [15]. A recently published study by Kosiborod et al. studied the effects of semaglutide by measuring the changes through the Kansas City Cardiomyopathy Questionnaires Clinical Summary Score (KCCQ-CSS), where an increase in the score highlights the physical capabilities and symptom improvement observed in heart failure patients. After the use of GLP-1 RAs, obese and diabetic patients, respectively, demonstrated a 16.6- and a 13.7-point improvement in the KCCQ-CSS scale. Additionally, patients significantly improved their daily walking ability compared to the control group [27,37]. Similarly, a trial assessing the variations in CVD in non-diabetic, obese patients showcased a 20% decrease in the risk of death after using semaglutide for 33 months [39]. These findings have highlighted the emerging effectiveness of this class of medication.

Cardiac outcomes of individual GLP-1 RAs in light of CVOTs

Numerous CVOTs have been conducted over the years, signifying significant benefits of GLP-1 RAs, with overall improved cardiovascular health and kidney function recorded [40]. A review by Helmstädter et al. in 2021 outlined these benefits, with liraglutide reducing death of heart-related disease events by 13% as per the LEADER trials and semaglutide demonstrating a 26% reduction in MACE. Similarly, the REWIND and HARMONY trials showed that dulaglutide and albiglutide also caused a noteworthy decrease in cardiovascular events. However, the ELIXA trial focusing on GLP-1 RAs could not record any significant benefit of lixisenatide [12].

Liraglutide

Mann et al. highlighted the effectiveness of liraglutide in patients with chronic kidney disease (CKD), where the primary cardiac outcome showed a hazard ratio of 0.69, recording a 31% lower risk of these events in patients with estimated glomerular filtration rate (eGFR) less than 60 mL/min/1.73m^2^ (p=0.05) [41]. However, Mosenzon et al., while elaborating on the added benefits of liraglutide in the reduction of MACE, noticed that this effect was more pronounced in patients with lower urinary albumin creatinine ratio (UACR), as more adverse cardiovascular events and all-cause death were reported in patients taking liraglutide who had UACR of >300 [42].

Dulaglutide

The REWIND trials, which studied a GLP-1RA named dulaglutide specifically, showed similar results, with a protective effect on heart health. Konig et al. in their post hoc analysis summarized that dulaglutide caused a significant reduction of 16.7% in HBA1c and 25.4% in UACR, which, in turn, contributed to the benefits recorded for cardiovascular health [43,44]. Following the promising results of the REWIND trial, FDA approved Trulicity (Dulaglutide) in those with T2DM who either have cardiovascular disease or are at increased risk of the disease to be used preventively for reduction in MACE [45].

Semaglutide

Semaglutide is a drug used subcutaneously and orally. It has yielded benefits as a weight-loss medication. Various CVOT trials, such as the SELECT, SUSTAIN, and SOUL trials, have demonstrated varying effects on heart diseases over the years [13,39,46]. As mentioned by Lingvay et al., semaglutide has shown promise as a treatment option for non-diabetics with established atherosclerotic disease by reducing their risk of MACE by 20% despite having varying HBA1c baselines [47]. Moreover, the SUSTAIN trials, which studied the effects of semaglutide in those with T2DM, also demonstrated significant improvement in risk factors, with a substantial 26% relative reduction in cardiovascular events such as nonfatal myocardial infarction and stroke [13,14,29]. These results showcase the ability of semaglutide to be a treatment option for cardiovascular benefit, notwithstanding the glycemic index. Likewise, the PIONEER trials established that between oral semaglutide and placebo, there was a 53% decrease in cardiovascular mortality. Following these initial results, the SOUL trial was commenced to specifically study the efficacy of oral semaglutide on cardiovascular health and find if oral semaglutide can be more beneficial than placebo. Although the trial is ongoing, specific predictor markers suggest its early termination due to the successful results obtained so far [48].

As analyzed by the recent meta-analysis of these trials, there is a 32% prevalence of heart-related diseases in T2DM patients. In light of this, GLP-1 RAs provide a high potential of being incorporated into diabetic patients' treatment plans, similar to SGLT-2 inhibitors, as they decrease MACE by 14%, macroalbuminuria by 26%, and the risk of all-cause mortality by 12% [15,49]. The FDA has approved Wegovy (semaglutide) as a treatment option for decreasing the risk of cardiovascular events in overweight or obese individuals, following the promising results of these trials [50]. Other GLP-1 RAs have also shown varying benefits in clinical trials and are summarized in Table 1.

**Table 1:** Summary of the CVS outcome trials, their primary endpoints, and study populations. ACS, acute coronary syndrome; CKD, chronic kidney disease; CVS, cardiovascular; eGFR, estimated glomerular filtration rate; GDF-15, growth differentiation factor 15; MACE, major adverse cardiovascular events; MI, myocardial infarction; NT-pro-BNP, N-terminal prohormone of brain natriuretic peptide

Trial Name/GLP-1 agonist	Results of clinical studies	Study time	Study population
LEADER: Liraglutide [18,28, 29,37,51]	13% reduction in MACE. The primary outcome of MI, nonfatal stroke, and CVS death was reduced (hazard ratio: 0.74, p < 0.001). For patients with kidney disease with eGFR < 60 mL/min/1.73m^2^, no significant benefit was recorded. The onset of kidney failure and major kidney disease was decreased by 24% at the end of 3.4 years.	Follow up 3.5-5 years	Patients with type 2 diabetes and HBA1c of 7% or more, who have not been treated with more than 2 oral diabetic drugs. Patients of 50 years or older with established CVS disease or CKD. Patients with eGFR < 60 mL/min/1.73m^2^ as per Cockcroft-gault formula. Type 2 diabetics with high-risk factors for CVS disease.
REWIND: Dulaglutide [43,52]	Reduction in the incidence of MACE (hazard ratio of 0.88). A 12% reduction in a patient taking dulaglutide as compared to the placebo. A 16% decrease in non-fatal stroke. 12% reduction in CVS mortality. Slower increase in cardiac biomarkers compared to placebo, mainly NT-pro-BNP and GDF-15.	5.4 years	50-year-old or older patients with type 2 diabetes. HbA1c of <9.5% with or without a stable insulin regimen. Diabetic patients stable on oral antihyperglycemic drugs. Patients with at least two CVS risk factors or a history of previous CVS events. Patients with BMI > 23 kg/m^2^.
SELECT: Semaglutide [9,13,47]	The primary outcome of a decrease in nonfatal MI or stroke recorded a hazard ratio of 0.74, p = 0.001, for noninferiority was established. Reduction in major CVS events by 20% compared to placebo. The risk of CVS death was similar between Semaglutide and placebo, p=0.92.	2.1 years	Patients > 45 years with type 2 diabetes. Patients with HbA1c of 7% or more. Patients with established CVS disease. Patients with a BMI of 27 kg/m^2^ or higher. Patients with chronic heart failure (New York Heart Association class 1 or 2). Patients with CKD of stage 3 or higher and with at least one CVS risk factor.
SUSTAIN: Semaglutide [13,14,29]	Decrease in nonfatal MI, stroke (hazard ratio 0.74). In patients with no history of CVD, no difference in primary outcome was recorded (HR=1).	-	Age 50 years or older patients with type 2 diabetes. Similar criteria to the above-mentioned trials such as LEADER and SELECT.
PIONEER: Oral Semaglutide [29,53,54]	Oral semaglutide does not pose a greater CVS risk than placebo. MACE reduction by 21% with assessing for non-inferiority.	1.25 year	Diabetic patients 50 years or older with established heart disease, or patients with stage 3 CKD or higher. Patients with one or more CVS risk factors.
SOUL: Oral Semaglutide [35]	Primary outcome: time in the incidence of nonfatal MI, nonfatal stroke, and CVS death. Trial is ongoing and results are expected in the coming years	Ongoing: 5.5 years	Type 2 diabetic patients with established CVS disease, CKD, or established cerebrovascular disease.
ELIXA: Lixisenatide [26,55]	No statistical significance could be achieved and no benefit was recorded on CVS outcome after the use of lixisenatide. A 4-point decrease in MACE 1.02 (0.89-1.17).	2.1 years	Diabetics, with a recorded history of ACS. HbA1c 6.5%-10%.
AMPLITUDE: Efpeglenatide [49,56]	Significant decrease in the risk of heart failure by 39%. MACE reduction (hazard ratio: 0.73, p < 0.001). Decrease in eGFR, pulse pressure, and risk of composite renal outcome.	1.81 years	Type 2 diabetics with HBA1c of 7% or higher. Patients with CVS disease or risk factors. Patients with previously recorded CVS event. Patients with CKD or evidence of renal disease.

Effect of GLP-1 RAs on cholesterol in the human body

Cholesterol is an essential nutrient in an everyday diet as it is an essential component of eukaryotic plasma membranes, is a precursor to steroid hormone biosynthesis, and is essential in the biosynthesis of bile acids and vitamin D [57-59]. Despite this, cholesterol also plays a key in the pathogenesis of atherosclerosis, an underlying cause of coronary heart disease and CVD [60]. Atherosclerosis can develop in vital blood vessels in people as young as 15 years and increase in prevalence and extent with age up to 34 years [61]. Research has shown that the use of GLP-1 RAs can modulate cholesterol metabolism, affecting LDL cholesterol (LDL-C), HDL cholesterol (HDL-C) levels, and total cholesterol (TC) levels in patients with cardiometabolic diseases.

Mechanisms by which GLP-1 RAs modulate cholesterol metabolism

Cholesterol metabolism is essential in maintaining lipid homeostasis and cardiovascular health. While traditional therapies such as statins suppress the functioning of 3-hydroxy-3-methylglutaryl-coenzyme A (HMG-CoA) reductase, recent attention has turned toward GLP-1 RAs’ effects on modulating lipid profiles. Understanding these mechanisms could help manage dyslipidemia and cardiovascular risk [62].

Yao et al. found that GLP-1 can modulate cholesterol synthesis and homeostasis via the upregulation of ABCA-1 and downregulation of miR19b in isolated hepatocytes [63]. Similar evidence from another study in 2018 showed that GLP-1 RAs modulate metabolism similar to statins by suppressing HMG-CoA reductase and SREBP-1C (sterol regulatory element-binding protein 1C) [64]. This evidence is backed up by a study conducted by Hasegawa et al., which found that GLP-1 administration improves dyslipidemia by inhibiting HMG-CoA reductase in T2DM patients [65]. These findings suggest that GLP-1 RAs may positively modulate cholesterol metabolism and dyslipidemia beyond their antidiabetic effects.

Effect of GLP-1 RAs on LDL-C, HDL-C, and TL levels

Studies have shown contradictions regarding the effect of GLP-1 RAs on LDL-C levels in the body, specifically with liraglutide or exenatide. Some studies found that LDL-C levels significantly reduced following treatment with liraglutide 1.2 mg/day or 1.8 mg/day (-0.28 and -0.23 mmol/L), exenatide once weekly (-0.13 and -0.17 mmol/L), and exenatide twice daily (-0.25 mmol/L and -6% change from baseline) [66]. Similarly, a study found a significant reduction in LDL-C following treatment with liraglutide 1.8 mg/day (-0.44 mmol/L) and exenatide 10 µg twice a day (-0.40 mmol/L) [51]. However, a study conducted by Diamant et al. found that the use of exenatide once weekly has nonsignificant effects on LDL-C levels with a change of -0.05 mmol/L ± 0.05 mmol/L, suggesting that the effect of GLP-1 RAs on LDL-C levels requires further research [67].

Similar to research on LDL-C levels and how the use of GLP-1 RAs such as liraglutide and exenatide can affect it, reports found that liraglutide and exenatide treatment also has insignificant effects on HDL-C [66-68]. Nonsignificant changes in HDL-C levels (-0.04 to 0.00 mmol/L) were reported following liraglutide treatment [66]. This study was corroborated by another study by Diamant et al., where HDL-C levels nonsignificantly changed from the baseline following treatment with exenatide once weekly (0.00 mmol/L) [67]. In contrast, a study found that liraglutide treatment once a day can show a significant reduction in HDL-C (-0.04 mmol/L, p<0.05) [51]. A meta-analysis in 2016 including 34 papers that measured changes in HDL-C levels before and after treatment with a GLP-1 RA found five studies showing a significant increase in HDL-C levels and 29 studies showing no significant change in HDL-C levels [69]. This disparity between results and the small proportion of studies that show statistical significance suggests that further research regarding the effect of GLP-1 RAs on HDL-C is also needed.

Unlike LDL-C and HDL-C, more studies found that GLP-1 RAs such as liraglutide and exenatide significantly decrease TL [51,67,68]. Administering liraglutide once a day found a significant reduction in TL from the baseline (-0.20 mmol/L) [51]. This finding is further reinforced by studies that found significant reductions in TL using exenatide once weekly (-0.12 mmol/L), treatments using 10 μg of exenatide per day (-0.36 mmol/L), and treatments including exenatide versus placebo, liraglutide 1.8mg/day versus placebo, taspoglutide versus placebo, and many more studies suggested a significant reduction in TL when compared (p<0.05) [11-13,65-67]. Despite this, a meta-analysis in 2016 found that 19 out of 21 studies that measured the effect of GLP-1 RA against a comparator found no significant changes in TL [69]. A summary of all clinical trials mentioned can be found in Table 2.

Comparison of GLP-1 RAs to other cholesterol-lowering medications

Dysregulation of cholesterol can lead to atherosclerosis and CVD [60]. Statins inhibit the HMG-CoA reductase enzyme crucial for the hepatic biosynthesis of cholesterol [62]. As mentioned earlier, GLP-1 RAs function similarly to statins by inhibiting HMG-CoA reductase, suggesting that GLP-1 RAs can be prescribed as not only an antidiabetic medication but also as a cholesterol-lowering medication that can reduce the prevalence of CVD [64,65,70-73].

One benefit of GLP-1 RAs is that they do not cause hypoglycemia, either when used alone or in combination with metformin or thiazolidinediones [74,75]. On the other hand, the most common symptom associated with GLP-1 RAs is gastrointestinal symptoms, mainly nausea. Other common side effects include headaches, injection site reactions, and nasopharyngitis, but these do not usually result in discontinuation of the drug [74-77]. However, many studies fail to establish a causative relationship between treatment and symptoms, suggesting that GLP-1 RAs have a favorable safety profile.

The most common side effect of statin is statin-associated muscle symptoms (SAMSs), reported by 10% to 25% of patients receiving statin therapy. Furthermore, in an internet survey of former statin users, 60% reported SAMSs, and 62% reported discontinuation of statin due to side effects [78]. In addition, it was observed that those who took less than 80% of their prescribed statin therapy versus patients who completed their therapy had a 45% increase in all-cause mortality and a 15% increase in CVD events, suggesting that completion of therapy is crucial [79].

Thus, GLP-1 RAs offer a more-than-viable substitute to other standard cholesterol-lowering therapies, such as statins, as they function by modulating cholesterol metabolism similarly but are associated with less severe and less prevalent side effects [75,76].

Limitations

Lifestyle management is the first treatment for almost every disease. However, lifestyle measures alone are less effective in maintaining adequate weight loss over time and must be augmented with weight loss medication for better outcomes and sustainability over prolonged periods. GLP-1 agonists have appeared to be superior to lifestyle management alone for losing weight and have better patient attrition rates. Patients relying solely on lifestyle treatment for obesity tend to lose morale swiftly and give up on their healthy diets and exercise and revert to unhealthy food habits and sedentary lifestyles in the light of not being able to see adequate results soon enough, which sometimes make their health outcomes even worse.

GLP-1 agonists have demonstrated significant health benefits in controlling weight, blood glucose, blood pressure, and MACE and continue to show promising outcomes in different clinical trials and meta-analyses. However, social determinants of health pose a significant risk regarding their use in various populations, especially among the economically disadvantaged. The low-income groups, with minimal education, and certain racial/ethnic groups who have a higher burden of chronic diseases such as obesity, T2DM, and cerebrovascular and cardiovascular disease seem to be significantly constrained in using these medications. The factors that contribute to this behavior include poor or no health insurance coverage, limited access to newer medication, financial distress, poor health education and communication, and physician bias.

Insurance coverage for GLP-1 agonist medication or incretin-based therapies for weight loss to provide effective long-term patient care takes precedence. At the same time, they may be covered by insurance for T2DM, but they should be covered for other chronic diseases such as obesity. Obesity is a chronic disease that exacerbates the risk of severe health conditions (as mentioned above) and is the leading cause of all-cause mortality. However, insurance coverage for anti-obesity medications is restricted or unavailable. While some patients might afford to pay the total price out of pocket, others might have to prioritize buying the medicines covered by insurance for other health conditions. This is where one needs to advocate for insurance coverage of these drugs and to lower the prices as much as possible to achieve lasting patient compliance and help serve our patient communities better [80]. This will also help the insurance companies in the long run since taking these anti-obesity medications will significantly improve the patient's overall health and lower the risk of complications caused by obesity.

Hence, government officials, pharmaceutical companies, physicians, and public health advocates need to come forward to collaborate and make an effective policy on the pricing, insurance coverage, manufacturing, distribution, and availability of these medications for the greater good of the people.

Adverse effects

Since the GLP-1 agonists (semaglutide) have been approved for weight loss therapy, a new era for obesity management has developed rapidly. However, the drug regulatory authorities have also documented rare and occasional severe side effects associated with using GLP-1 agonists, especially GI disease. A recent publication from JAMA on the analysis of people who have been taking GLP-1 agonists from 2006 to 2020 shows that people who are taking GLP-1 agonists have a nine-fold higher risk of developing acute pancreatitis compared with the older drug for weight loss, bupropion, and four times more frequent intestinal obstruction and three times more frequent gastroparesis. However, the absolute risk for the abovementioned complications was less than 1% per year of use. However, there was no indication of risk for developing biliary disease. The wide use of these drugs and their adverse effects, although rare, need to be considered and calculated by patients who are using the drugs for weight. With that being said, the risks and benefits for those who use the drugs for weight loss would differ from that for people who are using them for the management of diabetes.

Further potential of GLP-1 RAs

Osteoarthritis

Osteoarthritis (OA) is a condition where the entire joint undergoes wear and tear, leading to the loss of cartilage, swelling in the joint lining, and thickening of the bone under the cartilage, which may cause the formation of bone spurs. These changes can cause pain and stiffness. Changes in the fat and nerve tissues within the joint can also contribute to the development of OA. So far, there have yet to be any direct studies in humans to show whether GLP-1-based treatments are effective for OA, pointing to a gap in current research. However, early animal studies and lab research data show promise [81].

A mix of disrupted biological processes, such as oxidative stress, problems with cell repair, aging cells, and the release of inflammatory molecules, causes OA. These processes activate the nuclear factor kappa-light-chain-enhancer of activated B cells (NF-κB) pathway and increase the production of enzymes that break down cartilage, such as MMP-3, MMP-13, ADAMTS4, and ADAMTS5.

Recently, researchers have started studying the role of the GLP-1 receptors in cartilage cells, though more research is needed. GLP-1 receptors have been found in both healthy and OA-affected cartilage cells in rat knees. Its signaling helps protect cartilage by reducing cell death, lowering inflammation, and preserving the joint's structure [82].

The synovium can change early in OA, even before cartilage breaks down. This includes the thickening of the synovial lining due to the immune cells' release of inflammatory substances [83]. Macrophages, a type of immune cell, can become either M1, which promotes inflammation, or M2, which helps reduce it.

GLP-1 receptors exist in both human and mouse macrophages [84,85]. When GLP-1 receptors are activated, they influence the balance between two macrophage pathways, promoting the M2 anti-inflammatory type [84,86]. This switch is essential in inflamed synovium because it lowers inflammation markers such as IL-6 and tumor necrosis factor-alpha (TNF-α) [87].

GLP-1 therapies may also help reduce macrophage accumulation in inflamed areas by blocking proteins that allow these cells to stick [88]. For instance, liraglutide has been shown to reduce oxidative stress in macrophages through GLP-1 receptor signaling [89].

In osteoporosis, GLP-1 RAs such as exendin-4 can help by increasing osteocalcin levels, a protein essential for bone health. They also improve the balance of other proteins, OPG and RANKL, which support bone formation and protection [90]. This means that GLP-1 could help treat osteoporosis and similar bone issues, particularly in people with T2DM.

Headache

GLP-1RAs are linked to reduced cerebrospinal fluid secretion and intracranial pressure due to their action on receptors in the choroid plexus, where they raise cyclic adenosine monophosphate (cAMP) levels and inhibit the Na+/K+ ATPase pump [91]. In a study with 39 participants (BMI ≥ 30 kg/m^2^), GLP-1RAs such as semaglutide and liraglutide, combined with standard weight management, led to significant weight loss and fewer headache days compared to controls, with lower acetazolamide doses [92].

A separate double-blind study assessed exenatide's effect on intracranial pressure in women with idiopathic intracranial hypertension [93]. Participants receiving exenatide had reduced intracranial pressure at 2.5 hours, 24 hours, and 12 weeks versus the placebo. This group also experienced a notable reduction in headache frequency. However, BMI remained unchanged, indicating that the intracranial pressure effect was likely a direct action of exenatide rather than weight loss alone.

Neuropathic Visceral Pain and Irritable Bowel Syndrome

Intrathecal administration of GLP-1 RAs, such as GLP-1 and exenatide, has reduced pain sensitivity in animals. These treatments helped alleviate hypersensitivity in various pain models, including those for formalin-induced, nerve injury-induced, bone cancer-induced, and diabetes-induced pain in both mice and rats [94].

In a study using rats with spinal nerve ligation, exenatide was found to reduce neuropathic pain, specifically by lowering sensitivity to touch (allodynia) [95-97]. The treatment appeared to counteract the abnormal expression of 591 genes in the spinal cord that had been altered by nerve damage, especially those involved in inflammation, including TNF-α and toll-like receptors. In another experiment with a similar model, exenatide injections into the spinal area decreased responses to heat and touch by increasing the spinal release of β-endorphins, IL-10, and IL-4, associated with reduced pain perception [98,99].

Morroniside, another GLP-1 RA, reduced pain responses in a dose-dependent manner in rats experiencing neuropathic pain. The effects peaked within an hour and lasted over four hours [100]. Daily administration of morroniside for a week showed consistent pain-relieving effects without developing tolerance. Another study showed that morroniside increased the production of IL-10 and β-endorphin genes in the spinal lumbar regions and cultured microglia of rats with nerve injuries [99].

Geniposide, an active compound in Gardenia jasminoides with GLP-1 receptor activity, also reduced pain caused by formalin in rats, and this effect did not lead to tolerance with repeated use [101]. Lamiophlomis rotata, a Tibetan herb containing iridoid glycosides with GLP-1 receptor activity, effectively relieved pain from formalin injections, nerve injuries, and bone cancer in rats [95]. When given in doses between 130 and 250 mg/kg, this herb reduced pain by 50-80% without causing tolerance. Shanzhiside methyl ester, the main active component of Lamiophlomis rotata, also significantly reduced pain sensitivity in neuropathic rats over time without leading to tolerance [102].

In a diabetic neuropathy model in rats, liraglutide improved pain thresholds and reduced sciatic nerve damage. It normalized markers such as malondialdehyde, nitric oxide, IL-6, and specific matrix metalloproteinases while increasing superoxide dismutase and IL-10 in the affected nerves [103]. Another study found that liraglutide also reduced the activation of microglial cells in the brain's cortex and thalamus in diabetic rats by lowering the expression of NLRP3 protein, a marker of inflammation in brain microglia [104].

In a different diabetic neuropathy model, the combination of oral amitriptyline and subcutaneous liraglutide and a formulation combining both drugs showed significant improvements in pain and inflammation markers in the sciatic nerve [96]. PKF275-055, a compound similar to vildagliptin and an inhibitor of DPP-4, also improved nerve conduction and pain sensitivity by restoring Na⁺/K⁺-ATPase activity in diabetic rats [105].

Research shows that GLP-1 receptor-like signals are present in colon nerves and elevated in irritable bowel syndrome (IBS) patients, possibly explaining the presence of more nerve fibers in their colon [106]. In studies, GLP-1 and exendin-4 increased nerve growth in dorsal root ganglion neurons but did not affect pain-sensitivity receptors, suggesting a focus on motility [107]. In IBS rats, exendin-4 reduced stress-induced defecation and pain sensitivity through gut neurons [108].

Furthermore, exendin-4 lowered pain and serotonin in colon-sensitized rats with low GLP-1 levels [109]. After treatment, serotonin reuptake transporter (SERT) increased, while tryptophan hydroxylase-1 (TPH-1) decreased in the colon, showing that exendin-4 may reduce pain by regulating SERT and TPH-1 levels. In another study, liraglutide alleviated LPS-induced visceral pain in rats by decreasing intestinal inflammation and IL-6 in the colon through nitric oxide response [18].

Research on constipation-predominant IBS patients showed lower GLP-1 receptor levels and serum GLP-1 compared to controls, which correlated with more abdominal pain [110]. A GLP-1 analog, ROSE-010, was tested in IBS patients for pain relief, effectively increasing intestinal muscle movement [111]. ROSE-010 provided dose-dependent pain relief in trials, especially with a 300-μg dose, showing benefits as soon as 20 minutes after administration. Those with constipation experienced better relief than those with diarrhea. Most patients found ROSE-010 preferable to prior treatments, with female patients responding more effectively.

## Conclusions

GLP-1 RAs’ distinct and unique mechanism of enhancing insulin release in response to glucose, increasing satiety, and slowing gastric emptying can be attributed to the previously mentioned benefits of weight management and glycemic control. Heart health benefits are even of interest; trials show that GLP-1 RAs decrease inflammatory markers, improve endothelial function, and decrease arterial stiffness. Paramount cardiovascular outcome studies such as SUSTAIN and LEADER trials describe reduced MACE, signifying GLP-1 RAs as a valuable additive in cardiometabolic management.

GLP-1 RAs, originally developed to strengthen insulin secretion and manage blood glucose levels, have shown impressive benefits on the far side of glycemic control. GLP-1 RAs provide a multifactorial and practical approach to managing T2DM and related metabolic conditions, along with obesity, dyslipidemia, and hypertension. Clinical research and studies uniformly demonstrate their ability to contribute to notable weight loss, improve lipid profiles, lower blood pressure, and reduce cardiovascular risk. Nonetheless, while these agents are well-tolerated, they have some side effects, most importantly gastrointestinal, which warrant attention.
